# CD4 T Cell Responses in Latent and Chronic Viral Infections

**DOI:** 10.3389/fimmu.2013.00105

**Published:** 2013-05-13

**Authors:** Senta Walton, Sanja Mandaric, Annette Oxenius

**Affiliations:** ^1^Department of Microbiology and Immunology, School of Pathology and Laboratory Medicine, University of Western AustraliaNedlands, WA, Australia; ^2^Institute of MicrobiologyETH Zurich, Zurich, Switzerland

**Keywords:** CD4 T cells, persistent viral infection, effector functions, differentiation, regulation

## Abstract

The spectrum of tasks which is fulfilled by CD4 T cells in the setting of viral infections is large, ranging from support of CD8 T cells and humoral immunity to exertion of direct antiviral effector functions. While our knowledge about the differentiation pathways, plasticity, and memory of CD4 T cell responses upon acute infections or immunizations has significantly increased during the past years, much less is still known about CD4 T cell differentiation and their beneficial or pathological functions during persistent viral infections. In this review we summarize current knowledge about the differentiation, direct or indirect antiviral effector functions, and the regulation of virus-specific CD4 T cells in the setting of persistent latent or active chronic viral infections with a particular emphasis on herpes virus infections for the former and chronic lymphocytic choriomeningitis virus infection for the latter.

## CD4 T Cell Responses during Latent Persistent Viral Infection

### Introduction

**Figure 1:**
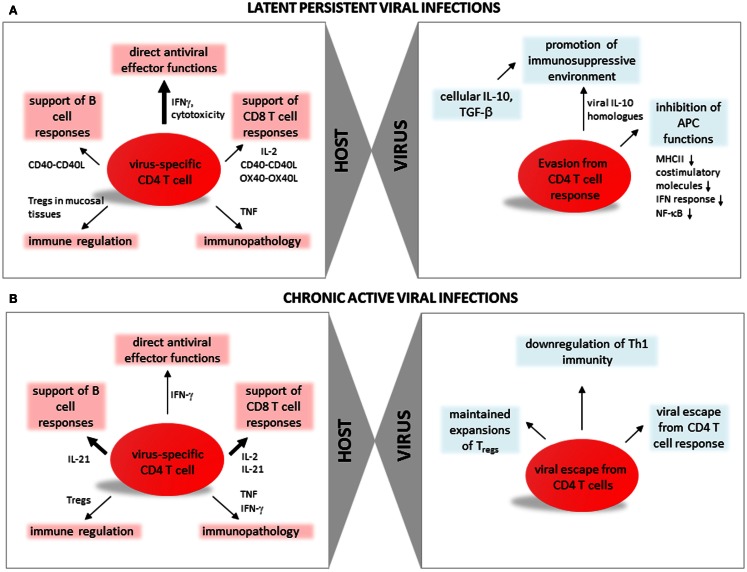
**CD4 T cell function, differentiation, and regulation during persistent viral infections**. **(A)** Latent persistent viral infections. In the left panel aspects of CD4 T cell immune responses with specificity for antigens of latent persistent viral infections are summarized: direct antiviral effector functions (discussed in paragraph Direct Antiviral Effector Functions of CD4 T Cells in Latent Persistent Viral Infections), support of CD8 T cell responses (CD4 T Cells Support Virus-Specific CD8 T Cells During Latent Persistent Viral Infections), support of B cell responses (CD4 T Cells Support Humoral Responses During Latent Persistent Viral Infections), immune regulation (Regulatory CD4 T Cells in Latent Persistent Viral Infections), and immunopathology (Role of CD4 T Cells in Latent Persistent Viral Infections and IL-10 Producing CD4 T Regulatory Cells). In the right panel viral strategies leading to escape from CD4 T cell responses are summarized (discussed in paragraph Viral Escape from CD4 T Cells). **(B)** Chronic active viral infections. In the left panel aspects of CD4 T cell immune responses with specificity for antigens of chronic viral infections are summarized: direct antiviral effector functions (discussed in paragraph CD4 T Cells Promoting Control of Chronic Viral Infection), support of CD8 T cell responses (CD4 T Cells Promoting Control of Chronic Viral Infections), support of B cell responses (Differentiation of CD4 T Cells During Active Chronic Viral Infections), immune regulation (T_regs_ and Chronic Viral Infections), and immunopathology (CD4 T Cell Mediated Pathology During Chronic Viral Infections). In the right panel viral strategies leading to escape from CD4 T cell responses are summarized (discussed in paragraphs CD4 T Cells Promoting Control of Chronic Viral Infection, Differentiation of CD4 T Cells During Active Chronic Viral Infections, and T_regs_ and Chronic Viral Infections).

Many viral infections are controlled by the host’s CD8 and B cell responses, leading to clearance of the infecting pathogen (acute/resolved infections). In this context CD4 T cells were mainly implicated to support CD8 T cell responses, especially during secondary infections, as well as in supporting B cells through their support of antibody class switch recombination and affinity maturation. However, in chronic latent infections as established by herpes viruses, CD4 T cells not only support CD8 T cells and B cells but exert direct antiviral effector functions that are crucial for terminating lytic replication and establishing viral latency, for controlling viral reactivation events, and finally to prevent morbidity and mortality of the host. Due to the generally low frequency of virus-specific CD4 T cells, this cell subset is far more difficult to study than their CD8 counterpart. Major advances in the study of chronic latent infections in recent years, however, unraveled not only the importance but also the phenotype and the functional capabilities of this T cell population. In the next paragraphs we will review the functions and phenotypes of CD4 T cells generated during chronic latent herpes virus infections with a special focus on *Cytomegalovirus* (CMV) (Figure 1A).

### Role of CD4 T cells in latent persistent viral infections

Human studies of primary immune deficiencies strongly indicate that CD4 T cells may be even more important than CD8 T cells in the control of herpes virus infections (Carneiro-Sampaio and Coutinho, 2007) (summarized in Table 1). In contrast to patients with compromised CD8 T cell functions, the susceptibility to viral infections, especially infections with the herpes virus family, was increased in patients with CD4 T cell deficiencies. The notion that robust CD4 T cell responses are beneficial for control of herpes virus infections is further supported by studies of chronically HIV infected individuals. HIV patients often suffer from herpes virus related disease due to frequent and uncontrolled viral reactivation. Patients with CD4 T cell counts below 100 cells/μl are at high risk to develop CMV-related disease (Gallant et al., 1992; Cinque et al., 1998) and CMV-seropositive HIV patients progress significantly faster to AIDS than their CMV negative counterparts (Webster et al., 1989; Sabin et al., 1995). Similarly, primary CMV infection in HIV patients, even with CD4 T cell counts >100 cells/μl, correlates with increased risk for earlier onset of AIDS (Robain et al., 2001). In a study of HIV-1-infected individuals loss of HCMV-specific CD4 T cells preceded CMV end-organ disease (Komanduri et al., 1998). Even in two patients with CD4 T cell counts above 400 cells/μl, recurrent CMV-related retinitis correlated with the loss of HCMV-specific CD4 T cells (Komanduri et al., 2001b). More recently, a correlation between low CD4 T cell counts and Kaposi sarcoma herpes virus (KSHV) DNA viremia was demonstrated in HIV infected individuals (Parisi et al., 2011). Low numbers of CD4 T cells in immune suppressed patients is further a risk factor for the development of EBV related disease (Sebelin-Wulf et al., 2007).

**Table 1:** **Role of CD4 T cells in herpes viral infections**.

Viral infection	Role of CD4 T cells			
	Establishment of viral latency/control of lytic viral replication	Maintenance of latency/inhibition of viral recrudescence	Immunopathology/immune senescence	Protective role in immunotherapy or immunization
HSV	Primary immunodeficiency (Carneiro-Sampaio and Coutinho, 2007); vaginal tract, sensory ganglia in mice (Milligan and Bernstein, 1997; Gill and Ashkar, 2009)		Immunopathology (Hendricks and Tumpey, 1990; Hendricks et al., 1992)	Protective role in vaccine models (Milligan et al., 1998; BenMohamed et al., 2003); major mediator of immunity in cornea (Hendricks and Tumpey, 1990; Hendricks et al., 1992)
VZV	Primary immunodeficiency (Carneiro-Sampaio and Coutinho, 2007); inverse correlation between viremia and virus-specific CD4 T cell response (Abendroth and Arvin, 2001; Malavige et al., 2008); SVV: (VZV infection model in rhesus macaques) prolonged viremia and higher viral titers in CD4 T cell depleted animals (Haberthur et al., 2011)	Positive correlation between CD4 T cell immunity and protection in the aged (Levin and Hayward, 1996; Weinberg et al., 2009) and in hematopoietic cell transplant recipients (Hata et al., 2002); increased reactivation in HIV carriers (Buchbinder et al., 1992)	Tonsillar CD4 T cells carry virus from the LN to the skin (Ku et al., 2002, 2004)	Protective role in transplant recipients with antiviral adoptive transfer treatment (Hata et al., 2002; Blyth et al., 2012)
MCMV	Particularly at mucosal sites (salivary glands) (Jonjic et al., 1989, 1990; Walton et al., 2011a)	Polic et al. (1998)		CD4 T cells isolated from CD8 deficient mice (Jonjic et al., 1990)
HCMV	Primary immunodeficiency (Carneiro-Sampaio and Coutinho, 2007); inverse correlation between viremia and virus-specific CD4 T cell response (young children versus adults) (Tu et al., 2004)	Reactivation in HIV patients with low CD4 T cell counts (Gallant et al., 1992; Cinque et al., 1998); loss of CMV-specific CD4 T cells (Komanduri et al., 1998, 2001b)	Graft-versus-host disease (Cray and Levy, 1993); myocarditis (Lenzo et al., 2002), atherosclerosis (Sacre et al., 2012), immune senescence (Karrer et al., 2009)	Transplant recipients (Einsele et al., 2002; Gamadia et al., 2003, 2004; Tormo et al., 2011; Akulian et al., 2013), septic shock patients (von Muller et al., 2007)
MHV-68	Particularly at mucosal sites (lung) (Christensen et al., 1999; Sparks-Thissen et al., 2005)	Cardin et al. (1996)		Adoptive transfer (Sparks-Thissen et al., 2005); immunization (Liu et al., 1999)
EBV	Primary immunodeficiency (Carneiro-Sampaio and Coutinho, 2007)	Reactivation in immunosuppressed patients with low CD4 T cell counts (Sebelin-Wulf et al., 2007)	Implicated in sustaining primary infection (Veronese et al., 1992; Fu and Cannon, 2000; Johannessen et al., 2000; Wagar et al., 2000; MacArthur et al., 2007)	Protective role in PTLD disorders following adoptive transfer of virus-specific CD4 T cells (Haque et al., 2007); murine model of PTLD-like and Burkitt’s lymphoma (Fu et al., 2004; Merlo et al., 2010)

The reconstitution of T cell immunity through infusion of *ex vivo* expanded virus-specific T cells in solid organ transplant patients undergoing herpes virus reactivation further confirmed the protective role of CD4 T cells. Adoptive transfer of VZV-specific T cells in hematopoietic cell transplant recipients undergoing VZV reactivation led to reconstitution of VZV-specific CD4 T cell responses and correlated with a reduced risk of VZV-induced disease (Hata et al., 2002; Blyth et al., 2012). Infusion of CMV-specific T cell lines restored HCMV-specific CD4 T cell immunity in stem cell transplant patients with CMV viremia which also correlated with reduced virus load (Einsele et al., 2002). Similarly, the presence of CMV-specific CD4 T cells correlated with virus control in renal or lung transplant patients (Gamadia et al., 2004; Akulian et al., 2013).

In renal transplant patients, early emergence of HCMV-specific CD4 T cells was an indicator for viral control (Gamadia et al., 2003; Tormo et al., 2011). In this study, a robust virus-specific CD4 T cell response preceded the CD8 T cell response in asymptomatic patients but not in patients suffering from HCMV-related disease. Further, as discussed in more detail in a later section, CD4 T cells were suggested to be important for the maintenance of functional antiviral CD8 T cells in several studies of transplant recipients (Walter et al., 1995; Einsele et al., 2002). Data from patients with post-transplant lymphoproliferative disorder (PTLD) indicate that transfer of EBV-specific CD4 T cells has a beneficial effect (Haque et al., 2007). These data are supported by murine models where CD4 T cells are efficient to control PTLD-like and Burkitt’s lymphoma even in the absence of CD8 T cells (Fu et al., 2004; Merlo et al., 2010).

Evidence that CD4 T cells are crucial antiviral effector cells in the control of herpes virus infections not only come from immunosuppressed patients but also from healthy individuals. Primary infection in children is accompanied with prolonged shedding of virus in the urine which was found to correlate with impaired induction of CMV-specific CD4 T cells but similar CD8 T cell responses compared to adults (Tu et al., 2004). In septic shock patients with an otherwise competent immune system, CMV recurrence was accompanied with an increase of HCMV-specific CD4 T cells and suppression of CMV replication was attributed to this T cell compartment (von Muller et al., 2007). Unfortunately, as HCMV-specific CD8 T cell responses were not analyzed concomitantly, the protective roles exerted by CD4 versus CD8 T cells remain elusive in these patients.

Findings in human studies of herpes virus infection correlate closely with data from mouse models. Although the acute phase of herpes virus infection can be controlled by redundant immune cell subsets involving CD8 T cells, B cell, and NK cells, CD4 T cells seem to have a crucial role in the establishment of viral latency and in the long-term control of the infection. For example, during MCMV infection, lytic viral replication cannot be controlled in the salivary glands in the absence of CD4 T cells and increased viral titers or prolonged viral replication can be detected in the spleen, lung, and liver (Jonjic et al., 1989, 1990). Further, CD4 T cells, in interplay with other immune cells, prevent viral recrudescence (Polic et al., 1998). In B cell-deficient mice direct antiviral effector mechanisms exerted by CD4 T cells were shown to be equally important to CD8 T cells for control of acute murine herpes virus (MHV)-68 replication (Christensen et al., 1999). Further, CD4 T cells are also essential for the control of HSV-2 replication at the site of infection, i.e., the vaginal tract (Milligan et al., 1998; Nakanishi et al., 2009), and in HSV-1 infection CD4 T cells are thought to be the major mediator of immunity in the cornea, but simultaneously may also be a major inducer of immunopathology (Hendricks and Tumpey, 1990; Hendricks et al., 1992).

The notion that CD4 T cells not only have a beneficial role was reported also for other herpes virus infections apart from HSV. The CD4 T cell subset may have deleterious effects in exacerbating collateral diseases such as myocarditis, immune senescence as well as graft-versus-host disease in CMV-seropositive patients (Cray and Levy, 1993; Lenzo et al., 2002; Karrer et al., 2009), and in EBV infection CD4 T cells, especially with a Th2 phenotype, may sustain primary infection via the expansion of EBV-transformed B cells (Veronese et al., 1992; Fu and Cannon, 2000; Johannessen et al., 2000; Wagar et al., 2000; MacArthur et al., 2007).

#### Direct antiviral effector functions of CD4 T cells in latent persistent viral infections

Although CD4 T cells were shown to have direct effector function in some acute resolved infections (Maloy et al., 2000; Brown et al., 2006, 2012), CD4 T cells are mainly thought to orchestrate other immune cells which then eradicate the infecting pathogens (summarized in Table 2). Their ability to produce a wide range of cytokines as well as their expression of various cell surface molecules implied in regulating immune functions (e.g., CD40L) in response – and importantly – adapted to different stimuli, endows them with the ability to modulate the immune response in a way that the most effective elimination of the causative agent is promoted. However, it becomes more and more apparent that although helper mechanisms are indeed a major function of CD4 T cells, this cell subset is also able to kill or inhibit pathogen replication in a more direct way during herpes virus infections (Stevenson et al., 2009). In recent years great advances were made to dissect exact antiviral mechanisms exerted by CD4 T cells during herpes virus infections, especially by using mouse models.

**Table 2:** **CD4 T cell effector functions in herpes viral infections**.

Viral infection	CD4 T cell effector function		
	Direct antiviral effector function of CD4 T cells	Support of CD8 T cell responses	Support of humoral responses
HSV	IFNγ (Chan et al., 2011)	Support of immigration into infected tissue via IFNγ-dependent mechanism (Nakanishi et al., 2009); support entry into draining lymph node (Kumamoto et al., 2011); priming of CD8 T cells and function (IFNγ and TNF secretion but not cytotoxicity) (Jennings et al., 1991; Rajasagi et al., 2009)	Antibodies mediate limited control (Chan et al., 2011)
VZV		Support CD8 T cells in SVV infected animals (Haberthur et al., 2011)	Protective role of passively transferred antibodies (Gershon et al., 1974; Zaia et al., 1983); support of isotype-switched antibodies in SVV infected animals (Haberthur et al., 2011)
MCMV	IFNγ (Jonjic et al., 1990; Lucin et al., 1992; Polic et al., 1996; Walton et al., 2011a)	Memory inflation and maintenance of functionality (Snyder et al., 2009; Walton et al., 2011b); help likely provided by IL-2 secretion (Walton et al., 2011b)	Support of isotype-switched antibodies (Jonjic et al., 1989); antibodies inhibit viral dissemination during acute viral replication and reactivation (Jonjic et al., 1994; Polic et al., 1998; Klenovsek et al., 2007; Wirtz et al., 2008)
HCMV	IFNγ (Sester et al., 2001; Gamadia et al., 2003; Nebbia et al., 2008); cytotoxicity (Tazume et al., 2004; van Leeuwen et al., 2004, 2006; Casazza et al., 2006; Delmas et al., 2007; van de Berg et al., 2008)	In transplant recipients (Walter et al., 1995; Einsele et al., 2002); in HIV patients (Komanduri et al., 2001a)	Administration of HCMV antibodies is beneficial in some cases (Yeager et al., 1981; Snydman et al., 1987; Fowler et al., 1992; Guglielmo et al., 1994; Messori et al., 1994; Harrison et al., 1995; Munoz et al., 2001)
MHV-68	IFNγ (Dutia et al., 1997; Sarawar et al., 1997; Sparks-Thissen et al., 2005; Tsai et al., 2011)	Progressive loss of virus-specific CD8 T cells in absence of CD4 T cell (Cardin et al., 1996); increased suppressive IL-10-producing and PD-1+ CD8 T cells in absence of CD4 T cells (Dias et al., 2010; Molloy et al., 2011)	Protective role of antibodies in lytic viral replication (Wright et al., 2009) and in recrudescence (Gangappa et al., 2002; Kim et al., 2002)
EBV	Soluble factors (Nagy et al., 2012); cytotoxicity (Khanolkar et al., 2001; Sun et al., 2002; MacArthur et al., 2007; Haigh et al., 2008)		B cell deficiency lack of viral persistence but also reservoir (Faulkner et al., 2000)

##### IFNγ

The observation that MCMV-specific CD4 T cells might directly protect the host via secretion of IFNγ was first reported by Lucin et al. (1992) as IFNγ neutralization and CD4 T cell depletion led to a comparable increase in viral titers in the salivary glands. In adoptive transfer studies, CD4 T cells isolated from MCMV-infected CD8 T cell-deficient mice dramatically decreased viral titers in sublethally γ-irradiated hosts in an IFNγ dependent manner (Jonjic et al., 1990; Polic et al., 1996). Although transfer of immune CD4 T cells was necessary for the observed protection, they were not sufficient, suggesting that CD4 T cells have to interplay with another cell type to confer protection. From this studies it was postulated that CD4 T cells cross-talk via IFNγ with other immune cells (e.g., NK cells in the salivary glands) to accomplish viral protection (Campbell et al., 2008). This hypothesis was challenged by recent results from our laboratory (Walton et al., 2011a). Using mixed bone marrow chimeras, we were able to verify that IFNγ secreted by CD4 T cells indeed directly controls MCMV replication in the salivary glands. IFNγ receptor (IFNγR) expression on irradiation resistant cells but not on immune cells was required for the control of viral replication, suggesting that IFNγ secreted by CD4 T cell signals either directly on infected non-hematopoietic cells or on bystander non-immune cells to confer protection and is not required for activation of other immune cells. The importance of IFNγ-secreting CD4 T cells in CMV was further supported by clinical data as higher levels of IFNγ-producing CD4 T cells correlated with faster viral control and reduced clinical symptoms (Sester et al., 2001; Gamadia et al., 2003; Nebbia et al., 2008).

The crucial role of IFNγ produced by CD4 T cells to inhibit herpes virus replication was also confirmed in the context of MHV-68 infection (Sparks-Thissen et al., 2005): IFNγ seems to act directly on myeloid cells where it suppresses viral lytic gene expression (Steed et al., 2007). However, the role of IFNγ in the control of latency is more controversial and likely strain dependent (Dutia et al., 1997; Sarawar et al., 1997; Tsai et al., 2011). In HSV-2 infection IFNγ-producing CD4 T cells seem to be essential to establish latent infection (Chan et al., 2011).

##### TNF

As treatment of MCMV-infected mice with recombinant IFNγ could not substitute for the lack of CD4 T cells, it was believed that CD4 T cells act via an additional mechanism to control viral replication. TNF was the most likely candidate due to the fact that IFNγ synergistically acts *in vitro* with TNF to inhibit viral replication (van der Meer et al., 1989; Pavic et al., 1993; Lucin et al., 1994). However, concomitant administration of recombinant IFNγ and TNF did not lead to the anticipated effect of enhanced viral control in the salivary glands in the absence of CD4 T cells (Pavic et al., 1993). *In vivo* administration of anti-TNF antibodies increased viral load in the salivary glands which led to the interpretation that neutralization of TNF abolishes the antiviral activities of CD4 T cells. However, in contrast to this hypothesis, when only CD4 T cells were deficient in TNF secretion, viral control in the salivary gland was comparable to wild type animals (Walton et al., 2011a). This demonstrates that CD4 T cells do not have to secrete TNF by themselves to cease MCMV replication in the salivary glands, but does not exclude that TNF secreted by other cells is crucial to confer protection in the salivary glands.

##### Cytotoxicity

Cytotoxic CD4 T cells during herpes virus infections were mainly studied in humans for a wide range of herpes viruses including HCMV, EBV, and VZV (Martorelli et al., 2010; Weinberg and Levin, 2010). Following primary HCMV infection a subpopulation of CD4 T cells emerges that is CD28 negative, expresses granzyme B, and maybe possesses cytotoxic capacities in renal transplant recipients (van Leeuwen et al., 2004). HCMV-specific CD4 T cells specific for pp65 showed surface mobilization of CD107a, a marker for degranulation, with concurrent loss of granzyme in response to *in vitro* restimulation (Casazza et al., 2006). Further, in the same studies, expression of granzyme A and B as well as perforin was highly elevated in terminally differentiated CD27^−^, CD57^+^ memory CD4 T cells, implicating that HCMV-specific CD4 T cells gain cytotoxic capacities with their increased maturation status. This attributed potential of cytotoxic activity of HCMV-specific CD4 T cells was verified by *in vitro* killing assays with antigen-pulsed EBV-transformed B lymphoblastoid cells (B-LCLs) incubated with CD4 T cells isolated directly *ex vivo* (Casazza et al., 2006; van Leeuwen et al., 2006). Using polyclonal CMV-specific CD4 T cell lines, Tazume et al. were able to demonstrate that these cells are able to kill HCMV-infected fibroblasts via a perforin-dependent mechanism as well as via Fas–FasL interaction. Further, direct cytolytic functions toward CMV-infected fibroblasts were dependent of IFNγ-induced upregulation of HLA-DR and required a 16 h incubation period (Tazume et al., 2004). These findings were challenged by a more recent study using IE1-specific CD4 T cell clones claiming that these cells are able to kill peptide-pulsed but not HCMV-infected target cells; although degranulation of CD4 T cells was measured after incubation with either target cells (Delmas et al., 2007). However, discrepancies in the incubation time of 16 versus 5 h could possibly explain this discrepancy. In accordance with the latter study, Tovar-Salazar et al. (2009) propose that HCMV-specific CD4 T cells fulfill a regulatory rather than cytotoxic functions. CD27^−^ CD28^−^ CD4 T cells sorted from HCMV-stimulated PBMCs of CMV-seropositive donors inhibited proliferation of autologous CMV-specific T cells in a dose-dependent manner, at least partly via secretion of TGFβ and granzyme B. Studies addressing cytotoxic CD4 T cells in the mouse model are very sparse (Jellison et al., 2005; Brown et al., 2006). However in mixed bone marrow chimeras that lacked perforin expression exclusively in CD4 T cells, MCMV replication was controlled in the salivary gland and in other organs comparably to their wild type counterparts (Walton et al., 2011a). This argues against a major role for cytotoxic CD4 T cells in MCMV infection; although cytolytic function via Fas–FasL interaction has not been addressed so far.

Granzyme, perforin, and granulysin expressing EBV-specific CD4 T cells were also documented *in vivo* and *in vitro* (Sun et al., 2002; MacArthur et al., 2007). *In vitro*, EBV-specific CD4 T cell clones exhibited cytolytic functions as assessed by the release of cytotoxic granules (Khanolkar et al., 2001; Sun et al., 2002) and to a lesser extent by Fas/FasL induced cell death (Wilson et al., 1998). Further, EBV CD4 T cell cultures expressing cytotoxic effector molecules (e.g., expression of granzymes, perforin, granulysins) inhibited long-term LCL growth (Haigh et al., 2008). Thus, CD4 T cells with cytolytic functions seem to be a hallmark of herpes virus infections.

#### CD4 T cells support virus-specific CD8 T cells during latent persistent viral infections

A role of CD4 T cells in providing help to CD8 T cells during CMV infection was already proposed a decade ago by studies carried out in HIV-1-infected patients (summarized in Table 2). In these individuals a strong HCMV-directed CD4 T cell response correlated with increased frequencies of HCMV-specific CD8 T cells recognizing an epitope of the pp65 ORF (Komanduri et al., 2001a). In human bone marrow transplant recipients, virus-specific CD8 T cells were able to protect from HCMV-related disease, however, their long-term maintenance depended on the presence of HCMV-specific CD4 T cells (Walter et al., 1995; Einsele et al., 2002).

More recently, the impact of CD4 T cell help on CD8 T cell responses was addressed in the context of MCMV infection by our group (Walton et al., 2011b) and others (Snyder et al., 2009). Lack of CD4 T cells impaired MCMV-specific CD8 T cell inflation [i.e., a continued accumulation of CMV-specific CD8 T cells over the course of CMV latency (Torti and Oxenius, 2012)], the maintenance of the memory CD8 T cell pool for the non-inflationary M45 and M57 epitopes, and the functionality of MCMV-specific CD8 T cells. MCMV-specific CD8 T cells isolated from a CD4 T cell-deficient environment displayed a more activated phenotype, indicating more frequent reencounter with their cognate antigens in the context of CD4 T cell deficiency. These observed differences were interpreted by Snyder et al. (2009) to be caused by the increased antigen burden seen in CD4 T cell-deficient MCMV-infected mice. However, when MCMV replication was controlled by interventional measures, it was revealed that in concomitant absence of ongoing replication and CD4 T cell help, MCMV-specific CD8 T cell inflation was completely abrogated. These data demonstrate that during MCMV infection (i) CD4 T cells are crucial for memory inflation of virus-specific CD8 T cells in the absence of overt viral replication, (ii) CD4 T cells support MCMV-specific CD8 T cells to remain functional, and (iii) long-term maintenance of non-inflationary MCMV-specific CD8 T cells is dependent on CD4 T cells.

Findings that CD4 T cells crucially influence MCMV-specific CD8 T cell responses were further underlined by results obtained by studying the role of OX40 (Humphreys et al., 2007b). The decreased CD4 T cell response in OX40-deficient MCMV-infected animals is believed to be responsible for the observed slight decrease in the inflationary CD8 T cell response at later stages of infection. Interestingly, during MCMV infection, exogenous treatment with anti-OX40 dramatically increased the CD4 T cell response as well as the virus-specific CD8 T cell response. However, MCMV-specific CD8 T cell responses were not influenced by OX40 treatment in MHCII^−/−^ mice, indicating that the increase of virus-specific CD4 T cells led to the increase in MCMV-specific CD8 T cells.

The available studies indicate a role for CD4 T cell-mediated supply of IL-2 to sustain CD8 T cell responses during the early or latent phase of herpesvirus infection (Jennings et al., 1991; Rajasagi et al., 2009; Walton et al., 2011b), or enhanced antigen presenting cells (APC) co-stimulatory functions for instance via CD40-CD40L (Sarawar et al., 2001), or OX-40-OX40L interactions (Humphreys et al., 2007b).

In HSV infection CD4 T cells enable CD8 T cells to immigrate into the infected tissue of the vaginal epithelium in an IFNγ dependent manner (Nakanishi et al., 2009). Recently, CD4 T cell support of CD8 T cell priming during early HSV-2 infection has been reported where CD4 T cells promoted the entry of naive polyclonal CD8 T cells to draining lymph nodes (Kumamoto et al., 2011), thus enhancing the priming of virus-specific CD8 T cells. Although MHV-68-specific CD8 T cell responses develop independently of CD4 T cell help (Belz et al., 2003), lack of CD4 T cells resulted in an increase of suppressive CD8 T cells which impaired MHV-68 control in an IL-10 dependent manner (Molloy et al., 2011). Furthermore, in the absence of MHC class II expression the inhibitory receptor PD-1 was significantly increased on CD8 T cells, resulting in higher viral titers in the lungs of MHV-68 infected mice (Dias et al., 2010). Interestingly, agonistic anti-CD40 treatment in CD4 T cell-deficient animals could rescue this phenotype.

#### CD4 T cells support humoral responses during latent persistent viral infections

Murine CMV-infected mice deficient in CD4 T cells lack isotype-switched and hence IgG antibodies directed against the virus (Jonjic et al., 1989) (summarized in Table 2). However, antibodies seem not to play an important role to control MCMV replication, as their absence did not impair control of lytic viral replication (Lawson et al., 1988; Jonjic et al., 1994). Nonetheless, MCMV-specific antibodies are able to inhibit viral dissemination during latency (Jonjic et al., 1994; Polic et al., 1998) and adoptive transfer of memory B cells into T and B cell-deficient RAG-1^−/−^ hosts conferred protection (Klenovsek et al., 2007). Further, in a more recent study, Wirtz et al. (2008) demonstrated that in addition to inhibit viral spread from the entry port, MCMV-specific antibodies are also able to locally inhibit dissemination at the site of viral replication. Administration of HCMV-specific antibodies to treat renal transplant patients with primary or reactivating infection was beneficial but this was not the case for all allogenic transplant patients (Snydman et al., 1987; Guglielmo et al., 1994; Messori et al., 1994; Munoz et al., 2001). Further, antibodies protect the fetus during pregnancy and premature infants in humans and in guinea pig models (Yeager et al., 1981; Fowler et al., 1992; Harrison et al., 1995). Evidence that CD4 T cells control at least partially MCMV replication via provision of B cell help come from studies of MHCII^−/−^ and CD4^−/−^ mice (Walton et al., 2011a). In contrast to MHCII^−/−^ mice, not only lacking CD4 T cells but also IgG antibodies, CD4^−/−^ mice, a mouse strain that is able to support immunoglobulin class switching, were able to eventually control MCMV replication in the salivary gland approximately 6 months post infection. This strongly indicates that CD4 T cells help B cells to produce neutralizing antibodies which seem to play a marginal role during the acute phase of infection but instead seem to be able to control MCMV replication during late stages of infection. Similarly, in MHV-68 infected mice, antibodies help to reduce dissemination of virus undergoing recrudescence from latency (Gangappa et al., 2002; Kim et al., 2002) and passive antibody transfer can reduce lytic viral replication (Wright et al., 2009).

The influence of antibodies during HSV-2 infection is more controversial but several studies indicate that B cell-mediated immunity through antibodies is able to reduce viral replication and to reduce HSV-2 related disease (Chan et al., 2011).

### Differentiation of CD4 T cells during latent persistent viral infections

Generation of virus-specific CD4 T cell responses requires the differentiation of naive CD4 T cells into potent antiviral effector cells. Recognition of viral antigens via pathogen-recognition receptors induces a signaling cascade that activates APC and induces their migration to the draining lymph nodes, where the priming of naive CD4 T cells occurs. CD4 TCR recognition of viral antigens presented on MHC class II molecules expressed on activated APCs, provision of APC-derived co-stimulatory signals, and the presence of a specific cytokine milieu induce activation, proliferation, and maturation of virus-specific CD4 T cells. Polarization of virus-specific CD4 T cells toward specific CD4 T cell effector subtypes depends on specific cytokine signaling inducing the activation of distinct CD4 T cell transcription factors (Swain et al., 2012), on the duration of antigen exposure (Constant and Bottomly, 1997), and on the antigen presentation capacity of certain APC subsets (Iwasaki and Medzhitov, 2010).

CD4 T cells with specificity for viruses being able to establish latency are mainly IFNγ, TNF, and IL-2 producing Th1 cells (Milligan and Bernstein, 1995; Munz et al., 2000; Walton et al., 2008). Differentiation of naive CD4 T cells into Th1 cells in general requires the presence of the inflammatory cytokines IL-12, type I IFN, and IFNγ (Constant and Bottomly, 1997). However, the specific role of these cytokines in priming of virus-specific CD4 T cell responses during herpes viral infections is not entirely identified. VZV-specific Th1 polarization was shown to be dependent on type I IFN and independent of IL-12 induction (Yu et al., 2005). In contrast, the administration of IL-12 was shown to enhance Th1 mediated antiviral responses toward HSV and EBV (Sin et al., 1999; Popescu et al., 2003). Our own results suggest that the priming of MCMV-specific CD4 T cells may be dependent on IL-12 induction, since the neutralization of IL-12 led to decreased MCMV-specific CD4 T cell responses (Mandaric et al., 2012). In addition, we identified that presence of the anti-inflammatory cytokine IL-10, secreted by APCs and other cells during the early phase of mouse CMV infection (Mandaric et al., 2012), leads to suppression of CD4 T cell priming. Thus, it seems that a critical balance of virally induced cytokine signals determines the extent of virus-specific CD4 T cell priming and the strength of induction of antiviral immune response. In addition to the most prevalent Th1 CD4 T cells, IL-17-producing CD4 T cells were reported during certain persistent viral infections, such as HSV-2 and CMV (Arens et al., 2008; Suryawanshi et al., 2011), although in significantly smaller numbers compared to Th1 cells, indicating the heterogeneity of *in vivo* generated virus-specific CD4 T cell responses. The precise role of Th17 cells in persistent viral infections remains still elusive, especially in respect to their antiviral function. Nevertheless, current reports suggest that Th17 cells contribute to virus-induced pathology during persistent viral infections (Rajasagi et al., 2012).

Moreover, several studies addressed the role of co-stimulatory signaling pathways for the induction of CD4 T cell responses during herpes virus infections. The CD40-CD40L pathway was shown to induce protective Th1 responses against HSV-2 *in vivo* (Sin et al., 2001). Recently, the B7-CD28 pathway was reported to regulate the induction of MCMV-specific CD4 T cell responses (Arens et al., 2011), whereas OX-40-OX40L pathway was shown to be dispensable for the primary MCMV-specific CD4 T cell response, but important to regulate the maintenance of CD4 T cell memory responses (Humphreys et al., 2007b).

### Regulatory CD4 T cells in latent persistent viral infections

CD4 T cells exerting immunosuppressive functions have been reported during a number of persistent latent virus infections. These cells regulate the extent of virally induced inflammation and play a crucial role in prevention of virus-induced immunopathology. The generation of an immunosuppressive environment, on the other hand, facilitates virus-persistence and the deliberate induction of anti-inflammatory responses is a prominent target of immune evasion strategies employed by herpes viruses.

#### Foxp3^+^ T regulatory cells during latent persistent viral infections

CD25^+^ Foxp3 T regulatory cells (T_regs)_ play an important role in regulation of immune tolerance but also in regulating antiviral immune responses. Their prominent function is the suppression of virus-specific CD8 T cell responses, either by cell-to-cell-mediated interactions or via the production of the anti-inflammatory cytokines IL-10 and TGF-β. Several reports imply the importance of CD25^+^ FoxP3^+^ T_regs_ in herpes virus infections, especially in suppressing inflammatory responses at mucosal peripheral tissues. In HSV-1 infection, T_regs_ were found at inflammatory sites of HSV infected ganglia and corneal stromal lesions in HSV-induced stromal keratitis (Veiga-Parga et al., 2012), serving to prevent tissue damage and attenuating the extent of inflammation (Belkaid and Rouse, 2005). On the other hand, T_regs_ delay the antiviral immune response toward herpes viral infections and may promote viral persistence (Suvas et al., 2003). Furthermore, the presence of CD4^+^ CD25^+^ cells was shown to suppress the function of antiviral CD8 T cells upon HCMV infection *in vitro* in an antigen-independent manner (Aandahl et al., 2004). Another study reported *in vitro* a role of T_reg_ secreted TGF-β for suppression of MCMV-specific T cell responses (Li et al., 2010). Taken together, although the precise mechanisms of CD25^+^ Foxp3 T_regs_ function still remain to be identified for many herpes virus infections, these cells represent an important regulatory subtype that strongly impacts the virus/host balance during persistent virus infections.

#### IL-10-producing CD4 T regulatory cells

One potent mechanism of CD4 T cell exerted regulatory functions is the secretion of the immunosuppressive cytokine IL-10. Development of virus-specific CD4 T cells producing IL-10 has been reported for numerous herpes virus infections, such as VZV (Vukmanovic-Stejic et al., 2013), HSV (Ramakrishna et al., 2011), CMV (Humphreys et al., 2007a), and EBV (Marshall et al., 2003) infections. The secretion of IL-10 has been detected in different subsets of CD4 T cells, including FoxP3^+^ CD4 cells (Wingate et al., 2009; Vukmanovic-Stejic et al., 2013) or FoxP3^−^ CD4 cells (Marshall et al., 2003; Ramakrishna et al., 2011).

CD4 T cell-derived IL-10 is in general proposed to serve as an inhibitory mechanism to regulate the function of other immune cells, such as macrophages and APCs or IFNγ-producing CD4 T cells (Saraiva and O’Garra, 2010) and thus to suppress immunopathology inferred by activated immune cells. Recently, in an HSV-induced encephalitis model, IL-10 production by FoxP3^−^ICOS^+^ CD4 T cells was shown to suppress the pathogenic function of inflammatory macrophages in the brain (Ramakrishna et al., 2011). Moreover, the recovery from EBV-induced infectious mononucleosis has been correlated with the appearance of IL-10-secreting virus-specific CD4 T cells (Marshall et al., 2007). These CD4 T cells were specific for the latent EBV antigen LMP-1 and were shown to inhibit IFNγ secretion of CD4 T cells of other EBV antigen specificities. Furthermore, during the persistent phase of MCMV infection, IL-10-producing FoxP3^−^ CD4 T cells have been detected selectively in salivary gland mucosal tissue, which is a major site of mouse CMV persistence and hence an important organ for horizontal virus transmission (Humphreys et al., 2007a). Interestingly, the activation of CD40-CD40L pathway altered the ratio of IFNγ- to IL-10- secreting CD4 T cells in this mucosal tissue, suggesting that IL-10 may act to suppress the downstream effects of co-stimulatory-mediated signaling pathways.

### Viral escape from CD4 T cells

Regarding the pivotal role of CD4 T cells, herpes viruses encode an armor of different proteins to manipulate CD4 T cell responses (reviewed by Wiertz et al., 2007; Abendroth et al., 2010; Zuo and Rowe, 2012). Immune evasins target several aspects of CD4 T cell maturation and function (Powers et al., 2008; Mason et al., 2012), impair DC functions by down-regulating co-stimulatory molecules (Rolle and Olweus, 2009), by interfering with the IFN responses (Marshall and Geballe, 2009), by manipulating the NFκB pathway, by modulating expression of MHC class II (Wiertz et al., 2007) as well as co-stimulatory molecules (Loewendorf et al., 2004; Mintern et al., 2006), and even by encoding their own inhibitory receptors (Cheung et al., 2005). The MHC class II presentation pathway is targeted at several points (Wiertz et al., 2007; Abendroth et al., 2010; Zuo and Rowe, 2012): herpes virus proteins were shown to suppress the CIITA promoter, thereby inhibiting MHC class II transcription, to degrade mRNA, to target the folded MHC class II protein for degradation, and to manipulate the invariant chain of the MHC class II molecules, thereby interfering with proper loading and presentation of CD4 T cell epitopes. On the cell surface they can divert the MHC class II molecules to exosomes and manipulate their recognition by the T cell receptor (Ressing et al., 2003). A comprehensive review of mechanisms exploited by herpes viruses to evade recognition by CD4 T cells is far beyond the scope of this review but the vast abundance of proteins encoded by these viruses to evade this lymphocyte subset further underlines their importance and effectiveness for viral control.

Apart from encoding immune evasins, chronic viral infections induce an immunosuppressive environment, thereby promoting virus-persistence. A recent study in an experimental model of HCMV latency revealed that the secretome of latently infected cells is enriched in immunosuppressive cytokines such as IL-10 and TGF-β that inhibit CD4 T cell cytokine secretion and MHC class II-mediated CD4 T cell cytotoxicity, highlighting a new mode of evasion from CD4 T cell responses during HCMV latency (Mason et al., 2012). We have recently shown that early induction of cellular IL-10 during acute mouse CMV infection leads to impaired NK/DC cross-talk and suppression of CD4 T cell priming affecting CD4 T cell proliferation and cytokine secretion, thus shifting the virus/host balance and promoting virus-persistence (Mandaric et al., 2012).

Intriguingly, in spite of the large number of immune-regulatory functions of herpes viruses, a robust CD4 as well as CD8 T cell response can be elicited in the host and contributes to protection. One possible explanation is that viral antigens are not directly presented by infected cells but rather by bystander cells such as APCs. These cells may process and present viral antigens to T cells after taking up viral proteins, defective viral particles, or apoptotic bodies from CMV-infected cells without being necessarily infected themselves. As presentation of exogenous antigens to CD8 but not CD4 T cells requires cross-presentation, a function specifically confined to particular DC subsets, CD4 T cells may have advantages to respond to herpes virus infections especially in organs devoid of cross-presenting APCs. Consistent with this hypothesis, we recently could demonstrate in MCMV-infected hosts that salivary gland-resident APCs lack mechanisms to present exogenous antigens to CD8 T cells, which likely contributes to the dependence on CD4 T cells to control lytic MCMV replication in this organ (Walton et al., 2011a).

## CD4 T Cell Responses during Active Chronic Viral Infections

### Introduction

A major challenge which actively replicating chronic infections impose on host immunity is the continued encounter of high levels of viral antigens by adaptive immune cells, which requires that immune cell numbers and their function ought to be regulated to afford a certain level of virus control while avoiding detrimental immunopathology. Furthermore, rapid evolution and immune-mediated antigen escape variant selection in case of persistently infecting viruses with high mutation potential present a continuously evolving spectrum of new antigenic determinants to the host’s immune system. A complex regulatory network adjusts the size and the function of adaptive immune responses during actively replicating chronic infections. This regulation is particularly well understood for CD8 T cell responses which bear the potential to cause major immunopathological insult via direct cytotoxicity and proinflammatory cytokine production (Frebel et al., 2010; Wherry, 2011). Considerably less is currently known about the regulation and differentiation of CD4 T cell responses during active chronic viral infections. In this part of the review we will summarize current knowledge on this topic, focusing on the role of CD4 T cells and the differentiation and maintenance of CD4 T cells during active chronic viral infections, on the role of T_regs_ and means of manipulating CD4 T cell responses during active chronic viral infections (Figure 1B). We will concentrate on insights from the murine chronic lymphocytic choriomeningitis virus (LCMV) and Friend virus (FV) infection models and complement those with observations made in humans chronically infected with HIV-1 and Hepatitis C virus (HCV).

### Role of CD4 T cells in active chronic viral infections

#### CD4 T cells promoting control of chronic viral infections

Observations from a number of experimental or natural chronic active viral infections in mice and humans indicate that presence of CD4 T cells and in particular of functional virus-specific CD4 T cells is involved in control of the chronic infection. In human HIV-1 infection robust HIV-1-specific CD4 T cell responses, measured by their proliferative capacity, their functional avidity, their cytokine secretion potential, and their frequencies, are associated with low HIV-1 plasma viral loads (reviewed in Porichis and Kaufmann, 2011). Conversely, vigorous and functional CD4 T cell responses during the early phases of HCV infection correlate with subsequent control of the infection (reviewed in Klenerman and Thimme, 2012). More direct evidence for the importance of CD4 T cells responses in the control of chronic viral infections is available from the mouse experimental infection models of LCMV and FV.

##### LCMV infection

Infection of immune-competent mice with high doses of specific LCMV strains (i.e., LCMV Clone 13 and LCMV Docile) leads to protracted control of the infection and thereby results in a prolonged phase of active viral replication which lasts between 2 and 4 months for Clone 13 and Docile, respectively (Althage et al., 1992). This infection model has proven to be very useful to study the consequences of prolonged exposure of adaptive immunity to high levels of viral antigens and conversely has been used to elaborate interventions which allow altering the virus host balance in favor of virus control (reviewed in Klenerman and Hill, 2005; Frebel et al., 2010; Wherry, 2011). A number of seminal observations were first made in this experimental model which were later confirmed and extended to persistent human viral infections (Pircher et al., 1990; Seiler et al., 1999; Hangartner et al., 2006b; Frebel et al., 2010; Wherry, 2011). Most importantly, physical and functional down-regulation of virus-specific CD8 T cell responses (T cell exhaustion) was first described in LCMV infection (Moskophidis et al., 1993) and has since been a matter of intense investigation. A number of molecular pathways contributing to T cell exhaustion were identified over the past years (Frebel et al., 2010; Wherry, 2011) and this knowledge is currently used to develop interventions with the aim of restoring CD8 T cell numbers and function in the setting of chronic viral infections or in cancer therapies (Blank and Mackensen, 2007; Topalian et al., 2012).

Numerous reports indicated exacerbation of chronic LCMV infections in the absence of CD4 T cells (Battegay et al., 1994; Altfeld and Rosenberg, 2000; Wiesel and Oxenius, 2012), preventing eventual control of infection (Matloubian et al., 1994; Ou et al., 2001; Fuller and Zajac, 2003; Wherry et al., 2003a). Such exacerbation was associated with progressive functional deterioration and deletion of LCMV-specific CD8 T cell responses (Matloubian et al., 1994; Zajac et al., 1998; Kristensen et al., 2002; Fuller et al., 2004). However it is largely unclear from these studies whether there is a direct contribution of CD4 T cells to promote control of chronic LCMV infection or whether CD4 T cells contribute to viral control indirectly via support of CD8 T cell responses or via support of humoral immunity. The majority of studies addressing this issue used adoptive immunotherapy interventions and point toward an indirect role of CD4 T cells in supporting LCMV control. LCMV-specific CD4, CD8, or B cells were adoptively transferred into chronically infected hosts and their ability to reduce viral loads was assessed. Adoptive immunotherapy studies performed in LCMV carrier mice (i.e., mice infected either at birth or *in utero* with LCMV which exhibit T cell tolerance toward LCMV) indicated a role for CD4 T cells, B cells, and IFNγ signaling for the control of LCMV infection (Planz et al., 1997) and transfer of memory T cells purged LCMV infection in carrier mice (Lauterbach et al., 2006; Garidou et al., 2009). Also, functional exhaustion of LCMV-specific CD8 T cells during adoptive immunotherapy in LCMV carrier mice can be prevented by co-transfer of antiviral CD4 T cells and B cells which led to reduction of viral loads, thereby lowering the antigen levels and facilitating the maintenance of functional CD8 T cells (Hunziker et al., 2002a). Finally, indirect evidence for direct or indirect antiviral activity of LCMV-specific CD4 T cells originates from the observation that transfer of LCMV-specific CD4 T cells into persistently infected mice can select for LCMV escape mutants that bear alterations in the targeted LCMV-derived CD4 T cell epitope (Ciurea et al., 2001b). A recent report also indicated that adoptive transfer of LCMV-specific CD4 T cells re-invigorated functional competence of LCMV-specific CD8 T cells (i.e., proliferation and cytokine production) in chronically infected mice, which was further augmented by concomitant PD-L1 blockade (Aubert et al., 2011).

The mechanisms by which T helper cells promote CD8 T cell responses are only beginning to be understood and very little is currently known about their role in sustaining virus-specific humoral immunity (Fahey et al., 2011; Harker et al., 2011). Overall, there is limited knowledge about the role, regulation, and evolution of antibody responses during chronic viral infections. However as antibodies bear less immunopathological potential compared to cytotoxic T cells, emphasizing humoral immunity with concomitant down-regulation of antiviral T cell responses might represent an important strategy of how the host protects itself against harmful immunopathology.

Two cytokines, namely IL-2 and IL-21, are critical for the mechanism by which CD4 T cells support CD8 T cell responses during chronic LCMV infection (Bachmann et al., 2007; Elsaesser et al., 2009; Frohlich et al., 2009; Yi et al., 2009; Walton et al., 2011b). In contrast to acute/resolved infections where memory CD8 T cell maintenance is antigen-independent but dependent on the homeostatic cytokines IL-7 and IL-15 (Schluns et al., 2000; Wherry and Ahmed, 2004; Surh and Sprent, 2008), maintenance of CD8 T cells during actively replicating chronic infections is strictly dependent on antigen and increased cell turnover (Wherry et al., 2003b; Agnellini et al., 2007). An ability which seems to be supported by T helper cells and in particular by IL-21 secreted by CD4 T cells in the context of chronic antigen encounter (Elsaesser et al., 2009; Frohlich et al., 2009; Yi et al., 2009; Iannello et al., 2010; Yue et al., 2010). These initial studies in LCMV infection were later extended to HIV infection and IL-21-producing CD4 T cells were associated with improved control of HIV-1 infection and preserved CD8 T cell function in humans (Iannello et al., 2010; Yue et al., 2010; Chevalier et al., 2011; Williams et al., 2011). While these findings support a direct role for IL-2/IL-21 signaling on CD8 T cell maintenance during chronic LCMV infection, they do not exclude a concomitant role of these IL-21-producing CD4 T cells for humoral immunity as IL-21 is a hallmark cytokine of follicular T helper cells (T_FH_), as discussed later in Section “Differentiation of CD4 T Cells During Active Chronic Viral Infections.”

##### Friend virus infection

Friend virus is a retroviral complex of Friend murine leukemia virus (F-MuLV) and spleen focus-forming virus (SFFV) and leads to persistent infection of mice (Hasenkrug and Chesebro, 1997). A recent review discusses various facets of CD4 T cell immunity during FV infection (Nair et al., 2011). Specifically, FV-specific CD4 T cells were shown to mediate direct antiviral effects leading to reduced viral replication and reduced spread of FV to the erythroid lineage as well as to reduced induction of erythroleukemia (Hasenkrug et al., 1998). CD4 T cells specific for the FV envelope protein provide substantial protection against FV-induced disease in the absence of CD8 T cells and antibody responses (Pike et al., 2009). The mechanisms by which FV-specific CD4 T cells afford control of FV infections relies on their ability to produce IFNγ. This cytokine exerts a direct suppressive effect on viral replication and augments CD4 T cell-mediated cytolytic activity (Iwashiro et al., 2001b). Consistent with a protective role of CD4 T cells, their *in vivo* depletion during acute infection promoted viral spread as well as onset of erythroleukemia and resulted in reduced maintenance of FV-specific CD8 T cells as well as neutralizing antibody responses (Nair et al., 2010).

#### CD4 T cell-mediated pathology during chronic viral infections

Besides contributing to direct or indirect control of active chronic viral infections, virus-specific CD4 T cell responses also bear the potential of causing immunopathology, particularly in case of (abnormally) increased frequencies or in case of defective immune regulation.

##### LCMV infection

The presence of large numbers of LCMV-specific CD4 T cells in the setting of a chronic infection predispose to CD4 T cell-mediated immunopathology. When transgenic mice expressing a TCR specific for the immunodominant LCMV CD4 T cell epitope are infected with high doses of LCMV, they succumb to TNF-mediated cachexia and immunopathology (Oxenius et al., 1998; Hunziker et al., 2002b). In beta-2 microglobulin deficient mice CD4 T cells are responsible for a chronic wasting syndrome (Doherty et al., 1993) and also in immune-competent mice contribute to morbidity (Stamm et al., 2012). Further, vaccination can exacerbate CD4 T cell-mediated pathology: when beta-2 microglobulin deficient mice are infected with LCMV they exhibited CD4 T cell-mediated immunopathology in the brain which was augmented by previous immunization with recombinant Vaccinia virus, and the severity of immunopathology correlated with the number of IFNγ-producing CD4 T cells (Hildeman et al., 1997). Furthermore, CD4 T cells were shown to contribute to the destruction of the splenic architecture following LCMV infection by a CD4 T cell derived TNF and IFNγ-dependent mechanism, leading to subsequent immunodeficiency (Matter et al., 2006).

##### Friend virus infection

During FV infection CD4 T cells mediate immunopathology as evidenced by bone marrow pathology, anemia, and weight loss via local IFNγ production by FV-specific CD4 T cells (Antunes et al., 2008). FV-specific CD4 T cells and their capacity to precipitate immunopathology was found to be regulated by B cells, as antigen presentation by B cells protected from CD4 T cell-mediated immunopathology by shifting the balance toward T_FH_ generation at the expense of Th1 differentiation (Ploquin et al., 2011).

### Differentiation of CD4 T cells during active chronic viral infections

Viral infections are generally associated with the differentiation of IFNγ-, TNFα-, and IL-2-producing Th1 cells, driven by Type I IFN, IL-12, and IFNγ production provided by innate immune cells during viral infections. This fate determination, provided by inflammatory cytokines (also referred to as signal 3), is initiated by the respective cytokine receptors and the downstream signaling transducer and activator of transcription (STAT) proteins which lead to the induction of master transcription factors (T-bet and eomesodermin in case of Th1 cells). While fate determination of CD4 T cells has been extensively studied in case of acute infections (reviewed in Zhu et al., 2010), the following section will specifically focus on the differentiation of CD4 T cells during active chronic viral infections.

#### Lymphocytic choriomeningitis virus

Akin LCMV-specific CD8 T cell responses (Frebel et al., 2010; Wherry, 2011) it was initially suggested that virus-specific CD4 T helper cells may also be exhausted during chronic LCMV infection (Oxenius et al., 1998; Fuller and Zajac, 2003; Fuller et al., 2004; Brooks et al., 2005), exemplified by a progressive loss of Th1-related cytokine production (IL-2, TNFα, and IFNγ). Not only recently primed LCMV-specific CD4 T cells are functionally down-regulated during chronic LCMV infection, but also pre-established vaccine-induced LCMV-specific and bystander CD4 T cell responses (Mothe et al., 2007). In a mouse model of dosed exposure of CD4 T cells to cognate antigen by inducible antigen expression in DCs, impaired JUN phosphorylation upon TCR stimulation was found in CD4 T cells which had been exposed for prolonged periods to high levels of antigen (Han et al., 2010). Antigen removal led to partial restoration of function (proliferation) but only to some extent to restoration of IL-2 and TNF production.

Transcriptional profiling of LCMV-specific CD4 T cells isolated from chronically infected mice indicated analogies in the transcriptional regulation of CD4 and CD8 T cell down-regulation during persistent infection. Several genes coding for inhibitory receptors and transcription factors which are involved in the functional down-regulation of virus-specific CD8 T cells were similarly regulated in the CD4 T cell counterpart (*Pdcd1*/PD-1, *Lag3*/LAG3, *Cd244*/2B4, *Tnfrsf9*/4-1BB, *Batf*/BATF, *Prdm1*/Blimp-1) (Wherry et al., 2007; Shin et al., 2009; Quigley et al., 2010).

Also in case of HIV-1 infection molecules of the B7 family are found to participate in CD4 T cell regulation, in particular PD-1, CTLA-4, and Tim-3 (reviewed in Kaufmann and Walker, 2009; Simone et al., 2009; Porichis and Kaufmann, 2012). A large fraction of HIV-specific CD4 T cells were found to co-express multiple inhibitory receptors, including PD-1, CTLA-4, TIM-3, and co-expression of these receptors correlated with increased plasma viral load and *in vitro* blockade of PD-1 promoted HIV-specific CD4 T cell proliferation (Day et al., 2006; Kassu et al., 2010). Also in chronic HCV infection, HCV-specific CD4 T cells express PD-1 (Kasprowicz et al., 2008) and CTLA-4 and blockade of PD-1, IL-10, and TGF-β is efficient in restoring *in vitro* proliferation and cytokine production of HCV-specific CD4 T cells (Raziorrouh et al., 2011).

Recent work, however, showed that LCMV-specific T helper cells seem to preferentially differentiate into T_FH_ during chronic LCMV infection, likely providing continuing support for virus-specific humoral immunity and eventual control of the infection (Fahey et al., 2011; Harker et al., 2011). Concomitant to the reduction in virus load, CD8 T cell responses were improved with respect to numbers and function (Fahey et al., 2011). Also during chronic LCMV infection, IL-6 has recently been identified to be the key molecule acting on CD4 T cells during late stages of chronic infection (Harker et al., 2011). Signals via the IL-6 receptor on CD4 T cells drove their differentiation into T_FH_ cells in a Bcl-6 dependent manner. Increased numbers of T_FH_ cells were essential for germinal center formation, LCMV-specific antibody production, and subsequent viral control. However, the capacity of LCMV-specific CD4 T cells to support LCMV-specific humoral immunity during established chronic infection does seem to have its limits, as they fail to support the generation of antibody responses specific for emerging escape variants (Ciurea et al., 2001a).

Prolonged TCR stimulation in the context of chronic LCMV infection seems to facilitate T_FH_ generation, which is independent of B cells (Fahey et al., 2011). This is consistent with a previous study showing that prolonged CD4 T cell activation favors T_FH_ differentiation independently of B cells (Deenick et al., 2010) but in contrast to situations with much lower amounts of CD4 T cell stimulating antigens where B cells are required for the differentiation of T_FH_ cells (Johnston et al., 2009; Cannons et al., 2010). In accordance to chronic LCMV infection expansions of T_FH_ with HIV-1 specificity were reported in case of human HIV-1 infection (Lindqvist et al., 2012).

These observations reinforce a lot of interest in the role of antibody responses during chronic infections. Antibodies can critically contribute to reduce systemic viral spread by limiting infectious particles via their neutralization or opsonization (Burton, 2002; Bachmann et al., 2004; Hangartner et al., 2006a,b; Bergthaler et al., 2009). To date it is unclear whether and how B cells need to be continuously supported by T_FH_ cells during chronic infection. This deviation from a typical proinflammatory Th1 response to a B cell helper response might be a means by which the host tries to reach an optimal equilibrium between virus control and avoidance of immunopathology. That such emphasis on T_FH_ cell differentiation during viral chronicity is linked to optimizing antibody responses, is a very attractive hypothesis and demands further investigations.

#### Friend virus

In the case of FV infection the functional capacities of FV-specific CD4 T cells seem to be affected by exposure to high levels of antigen. FV-specific CD4 T cells were found to produce IFNγ and to contribute to reduction of viral loads up to 2 weeks post transfer into FV infected mice, but thereafter lost their antiviral activity (Nair et al., 2010). Comparable to LCMV infection (Fahey et al., 2011), FV infection also induces Th1 and T_FH_ differentiation but as opposed to chronic LCMV infection T_FH_ differentiation is promoted by B cells (Ploquin et al., 2011), perhaps due to lower antigen amounts perceived by FV-specific CD4 T cells compared to LCMV-specific CD4 T cells.

### T_regs_ and chronic viral infections

A CD4 T cell subset which is involved in immune regulation in the context of chronic antigen exposure is the T_reg_ subset. Induced or increased numbers of T_regs_ as observed during chronic infections leads to suppression of effector CD8 T cell responses (Belkaid and Rouse, 2005; Punkosdy et al., 2011). In response to chronic LCMV infection marked and sustained expansion of T_regs_ is observed which is driven by infection-induced expression of a retroviral superantigen encoded in the mouse genome (Punkosdy et al., 2011). So far there is limited knowledge on how these increased numbers of T_regs_ impinge on LCMV-specific adaptive immunity. Besides T_regs_ additional cell types were shown to regulate the size of the antiviral CD4 T cell response in the setting of a chronic LCMV infection. These include NK cells which were shown to kill activated virus-specific CD4 T cells and therefore to reduce the overall size of the antiviral CD4 T cell pool (Waggoner et al., 2012) and IL-10-producing DCs or T cells which downregulate LCMV-specific CD4 T cell responses (Brooks et al., 2006, 2010; Ejrnaes et al., 2006).

In the FV infection model, T_regs_ develop during the second week of infection and suppress CD8 T cell functions which is associated with impaired virus control (Zelinskyy et al., 2006, 2009). T_reg_ frequencies double in size in response to FV infection compared to naïve mice and these cells remain immunosuppressive also after adoptive transfer into new hosts (Iwashiro et al., 2001a). T_regs_ from FV infected mice suppress *in vitro* effector cell differentiation of naïve CD8 T cells via direct cell-cell contact and irrespective of TCR specificity of the CD8 T cells. Further, they also suppress the function of *in vivo* activated effector CD8 T cells (Robertson et al., 2006). Transient depletion of T_regs_ is sufficient to reinvigorate virus-specific CD8 T cell responses, thereby decreasing virus load (Dietze et al., 2011) and also ameliorating the antiviral activity of FV-specific CD4 T cells (Nair et al., 2010).

In human HIV infection a majority of studies also report increased T_reg_ frequencies which were proposed to contribute to down-regulation of HIV-specific immunity but also to participate in limiting overall immune activation and preventing immunopathology (reviewed in Rouse et al., 2006; Moreno-Fernandez et al., 2012). Also in chronic HCV and HBV infection T_regs_ are implicated to modulate virus-specific immune responses, thereby potentially promoting disease progression or preventing infection triggered immunopathology (reviewed in Manigold and Racanelli, 2007; Miroux et al., 2010).

Taken together, sustained expansions of T_regs_ are observed in a number of chronic viral infections, sometimes accumulating at the site of viral persistence. Two opposing roles can be attributed to these increased numbers of T_regs_, on one hand they might impair virus-specific immunity and thereby promote viral persistence and on the other hand they might represent an important means of the host to limit immunopathology during protracted viral infections.

## Concluding Remarks

A large body of studies investigating CD4 T cell differentiation and function during latent and chronic viral infections has revealed that these cells possess a remarkable number of different functional facets. These range from direct antiviral protective functions via production of cytokines or direct cytotoxic activity (mainly demonstrated for human herpes virus infections), their long-term support of CD8 T cell and B cell responses, their immune-regulatory function to their potential of causing immunopathology.

Evidence for a direct antiviral function of CD4 T cells during persistent viral infections comes from studies in CD4 T cell-deficient or compromised hosts. A pivotal role of CD4 T cells in effectively contributing to viral control is also supported by the observation that herpes viruses have elaborated an impressive number of immune evasion mechanisms which target the CD4 T cell response and by the observation of viral mutational escape from immunodominant CD4 T cell responses during active chronic viral infections.

In the context of persistent viral infection a tight regulation of T cell responses is essential as effector functions triggered by the continued presence of antigen might bear severe immunopathological potential. While we have seen over the recent years an exciting progress in understanding the regulation of virus-specific CD8 T cells in the setting of persistent and chronic viral infections, much less is known about CD4 T cells. Recent data indicate that CD4 T cells are not functionally down-regulated akin their CD8 T cell counterpart in the setting of chronic viral infection but rather deviate their differentiation toward the T_FH_ subset, thereby likely avoiding immunopathology and at the same time supporting humoral immunity. Thus, our understanding of the regulation and function of CD4 T cells during persistent viral infections definitively needs to be advanced to understand immune control of persistent infections and eventually to harness this population of cells for vaccine or interventional purposes in the context of persistent viral infections.

## Conflict of Interest Statement

The authors declare that the research was conducted in the absence of any commercial or financial relationships that could be construed as a potential conflict of interest.
